# Book Review: Second Language Learning Motivation in a European Context: The Case of Hungary

**DOI:** 10.3389/fpsyg.2021.704500

**Published:** 2021-05-31

**Authors:** Dan Li, Weina Ouyang

**Affiliations:** School of Foreign Languages, Beihua University, Jilin, China

**Keywords:** motivation, hungary, second language acquisition, second language, language learning

Being considered as one of the quintessential components of individual differences in relation to language learning, second language (L2) motivation has received significant momentum in second language acquisition (SLA) research because it impacts learners' use of L2 learning strategies, their interaction with classmates, their general proficiency, and their L2 maintenance skills. Equally importantly, demotivational, amotivational, and remotivational factors play decisive roles in the process of language learning and teaching, so they need to be meticulously identified in each idiosyncratic learning context to help not only learners but also teachers to stay motivated. Kata Csizér's monograph, entitled Second *Language Learning Motivation in a European Context: The Case of Hungary*, furnishes a rundown of L2 motivation research in the Hungarian context, which has demonstrated to be an excellent laboratory for this kind of research. This volume provides a glimpse into the theoretical research on L2 motivation, coupled with comprehensive information on vast L2 motivation studies in Hungary. Being cognizant of the interdisciplinary nature of L2 motivation, Csizér also integrates qualitative analyses of the most significant investigations with quantitative data on teachers' opinions about success in L2 learning. Consisting of five chapters, this thought-provoking monograph meticulously delves into the constructs of L2 motivation from the perspectives of learners and teachers and sets forth future paths for L2 motivation research.

In Chapter 1, Csizér, sets the scene by informing the readers of the Hungarian education system, syllabi, and curricula, arguing convincingly that “teaching and learning involve teacher-student and student-student interaction within the classroom setting and different types of L2 programs (p. 16).” The author elucidates that various course books are taught in Hungarian schools, and the books “did not always match the proficiency level of the students, which might be demotivating for both students and teachers” (p. 18), justifying the pressing need to scrutinize the role of L2 motivation in the Hungarian context.

Chapter 2 foregrounds that L2 motivation theories have largely been inspired by research in the field of psychology. Being informed by Dörnyei (2001) and Gardner (1985) theoretical postulations, the author lays the foundations for different theoretical underpinnings of motivation. Conceptualizing motivation as “the dynamically changing cumulative arousal in a person that initiated, directs, coordinates, amplifies, terminates, and evaluates the cognitive and motor processes whereby initial wishes and desires are selected, prioritized, operationalized and (successfully or unsuccessfully) acted out” (p. 25), Csizér declares that inner drive is the most important factor in learning. In the rest of the chapter, the author elaborates on the classic theories of L2 motivation and accentuates that self-confidence and self-efficacy, as two determinants of motivation, play a pivotal role in second/foreign language learning.

Putting a premium on the role of student motivation, Chapter 3 brings to the fore the significant role of language choice in the Hungarian education system and highlights how such individual-related variables as the Canadian influences, autonomy, emotions, and cognitive factors can have a bearing on L2 motivation. Besides, the author stresses the role of the milieu by reporting the results of one of her previous studies. This chapter seems enlightening in that it also elucidates the pivotal role of intercultural contact, demotivation, and special educational needs of learners such as dyslexic and deaf language learners regarding L2 motivation.

Inasmuch as the fact that learning outcomes and success are assessed from the point of view of language learners, literature on L2 learning motivation is replete with the surge of interest on language learners, echoing what Dörnyei (2019) argues by reiterating that L2 motivation researchers are motivated “in their interest in the personality/identity of the language learner” (p. 31). Besides, the author cogently argues that teacher's motivational profiles, as well as teachers' techniques to motivate their learners, should be taken into account. To bridge this gap, the penultimate chapter illuminates the role of *teacher* motivation, their dispositions, their attitudes, and experiences by sketching the conceptualizations and theoretical frameworks. The chapter also presents the results of Csizér (2020) empirical study to corroborate how substantial the role of teacher's motivation can be. The author concludes that “it is the love for the job that helps teachers develop professionally rather than cognitive variables” (p. 135).

The closing chapter recapitulates the preceding chapters and opens up some avenues for further research. The chapter recommends some research directions for both student- and teacher-focused L2 motivation. Investigating L2 motivation in a dynamic way within the paradigm of attribution theory, exploring the demotivational and remotivational processes from multiferous perspectives, and scrutinizing the role of various cognitive variables are some of the student-related directions for further research. Moreover, the author highlights that we need to move toward a theoretical model of language teacher motivation by running confirmatory studies in various educational contexts, and teachers should be encouraged to devise motivational strategies to pique students' interest and focus in the classrooms. The monograph closes its mission by calling for novelty in research methods on L2 motivation.

All in all, had the author added some first-hand empirical studies instead of reporting her previous research, the authors would have benefitted more. However, this book is theoretically comprehensive, pedagogically viable, and intellectually thought-provoking because it reconciles theory and practice by providing thorough, contextualized, and refreshing information on L2 motivation in the Hungarian context, so I am confident to mention that this book is beneficial for a wide array of scholars interested in the role of L2 motivation, including methodologists, curricula designers, researchers, teachers, and students.

## Author Contributions

DL took the lead in writing the manuscript. Both authors provided critical feedback and helped shape the research.

## Conflict of Interest

The authors declare that the research was conducted in the absence of any commercial or financial relationships that could be construed as a potential conflict of interest.
